# Staging of Prostate Cancer: Role of Multiparametric Magnetic Resonance Imaging in Different Risk Classes

**DOI:** 10.5152/tud.2023.22261

**Published:** 2023-07-01

**Authors:** Francesco Saverio Guerra, Laura Eusebi, Francesco Bartelli, Sara Cecchini, Enrico Paci, Giuseppe Guglielmi

**Affiliations:** 1 Department of Clinical and Experimental Medicine, Foggia University School of Medicine, Foggia, Italy; 2 Radiology Hospital “Carlo Urbani”, Jesi, Italy; 3 Diagnostic Imaging, Clinical and Interventional Radiology, IRCCS INRCA, Ancona, Italy; 4 Radiology Unit, “Dimiccoli” Hospital, Barletta, Italy.; 5 Department of Radiology, Hospital IRCCS Casa Sollievo della Sofferenza, San Giovanni Rotondo, FG, Italy

**Keywords:** Andrology, multiparametric MRI, prostatic neoplasms, urology

## Abstract

Using multiparametric magnetic resonance imaging, it is now possible to diagnose prostate cancer and categorize its risk. As it can accurately determine the extracapsular extension of the tumor, invasion of seminal vesicles, involvement of lymph nodes, and the potential presence of bone metastases, multiparametric magnetic resonance imaging plays a crucial role not only in the diagnosis but also in the local staging of prostate cancer. The patients with a history of negative biopsy/increasing prostate-specific antigen and the existence of further data supporting its use in biopsy-naive patients and active surveillance are the most blatant indications for multiparametric magnetic resonance imaging in guidelines. The traditional clinical examination, prostate-specific antigen tests, and systematic biopsy are all enhanced by multiparametric magnetic resonance imaging, which will miss certain cancers due to insufficient size or changes in tissue density. The use of multiparametric magnetic resonance imaging is expected to rise, and further advances in the method will be crucial for the secure adoption of targeted therapeutic ideas. Here, we give a succinct overview of multiparametric magnetic resonance imaging's application to the identification and risk classification of prostate cancer.

**Main Points**

- Multiparametric magnetic resonance imaging (mpMRI) has clinical relevance in the diagnosis as ecoguided biopsy especially if supported by a correct clinical classification of the patient and an adequate risk estimate.
- The use of wider surgical margins to reduce the rate of positive margins or nerve-saving surgery to reduce morbidity are 2 choices that can be made based on information provided by MRI, which also helps to improve diagnostic accuracy.
- The best protocol for studying prostate cancer combines a high-resolution T2W sequence acquired over 2 planes (at least one axial) with 2 functional sequences: a dynamic sequence following intravenous contrast agent Dynamic Contrast Enhanced (DCE) administration and a weighted diffusion image acquisition (diffusion-weighted imaging).
- The invasion of the seminal vesicles and extracapsular disease spread, as well as a high probability of a positive surgical margin (predisposing to recurrence), lymph node, and distant metastases, are all associated with a worse outcome. Therefore, it is evident that the information gleaned from imaging plays a significant role in the choice regarding the best course of treatment (radical prostatectomy, radiotherapy, or hormonal deprivation).
- The staging of patients at high risk using next-generation imaging methods like the whole-body-MRI or PET CT is excellent and enables a better layering of the patient, allowing for the selection of the most appropriate therapy.

## Introduction

In Europe, the most common male cancer, after skin cancer, is prostate cancer, with an incidence of 200 per 100 000 men/year and a rather high mortality rate.^1,2^

The diagnosis of prostate cancer is based on the evaluation of different parameters such as serum levels of prostate-specific antigen (PSA), rectal examination (DRE), and transrectal ultrasound-guided biopsy.^3^

In the past, magnetic resonance imaging (MRI) has never had a large relief in the diagnosis of prostate cancer; however, recent studies have shown that multiparametric MRI (mpMRI) has clinical relevance in the diagnosis as ecoguided biopsy, especially if supported by a correct clinical classification of the patient and an adequate risk estimate.^4,5^

In 2001, the routine use of MRI in pretreatment diagnosis and staging was rather controversial among urologists.^6^

In 2008, opinions about the use of MRI in the diagnostic process began to change, considering prostate MRI, along with rectal exploration, as a possible method of diagnosis.^7^

Since 2012, MRI of the prostate gland has been fully integrated into the instruments adopted for the staging of prostate cancer, also approved by urologists.^8^

In order to standardize image acquisition procedures and interpretation, the European Society of Urogenital Radiology published a set of guidelines and recommendations in 2012 under the name “Prostate Imaging Reporting and Data System (PI-RADS)”; this work underwent a number of revisions and modifications over time in collaboration with the American Society of Radiology until the final version of 2019 (PI-RADS v2.1).^9^

For years, prostate cancer staging has relied on Partin nomograms to evaluate extracapsular cancer extension, using PSA, rectal exploration, and Gleason score (GS). The nomogram of Partin combines tumor stage, GS, and PSA to predict the final pathological stage of the tumor (accuracy 70%-80%) in the preoperative stage. The Partin nomogram, however, proved inaccurate in-stage prediction in cases of PSA >20, as evidenced by the high percentage of organ-confined disease and negative lymph nodes.^10^

This strategy exaggerates the true severity of the disease. By identifying the location and severity of the illness, MRI can aid in the development of a surgical plan. Using wider surgical margins to lower the rate of positive margins or performing nerve-sparing surgery to reduce morbidity are 2 choices made possible by this knowledge. In fact, the use of MRI in staging is considered as a level of evidence 2B and a level of recommendation A by urological guidelines.^1^

Extracapsular extension of the tumor is associated with a higher risk of positive surgical margins, biochemical recurrence, metastatic disease, and lower survival after radical prostatectomy. Therefore, having the most accurate possible evaluation of extracapsular extension before surgery is of the utmost importance to optimize clinical decision-making.

Magnetic resonance imaging has better diagnostic accuracy for extraprostatic extension than clinical parameters and even greater accuracy when MRI is combined with clinical information to estimate extraprostatic extension. Moreover, with the increase of multimodal treatment strategies for prostate cancer, it is even more important to carefully stage before treatment.^11^

The objective of our review is to provide an overview of recent innovations in the use of mpMRI, the introduction of different systems of classification of extraprostatic disease, and its role in the classification of different risk classes.

### Clinical and Research Consequences

#### Staging of Prostate Cancer

Therapeutic management of the prostate cancer population is based on a classification and staging system provided by a set of patients with similar parameters and conditions.^12,13^

The American Joint Committee on Cancer devised the “Tumor, Node, Metastasis” (TNM) staging approach, which is most frequently employed and is associated with serum PSA level and Grade Group (GG) biopsy.^14^

The location of prostate cancer is categorized into 4 groups based on the recommendations of the European Urology Association.^12,13^ Groups T1 and T2 contain tumors that are only found in the prostate gland. These subcategories include instances of asymptomatic neoplasms that are incidental to transurethral resection because they are not clinically evident (T1a–T1b) and clinically and radiologically significant (T1c-T2) in accordance with the sample's histological results.^14^ Locally progressed cancers that invade seminal vesicles and locoregional tissues fall under subcategories T3 and T4. Table 1 provides a detailed breakdown of the distinct traits of prostate cancer according to the TNM staging method.^14^

The GS is a classification method used in clinical practice to rate the aggressiveness of prostate cancer. It is decided by assigning a score (ranging from 1 to 5) to each of the two tumor parts that most accurately represent the tumor. The number 1 will be given to tumor tissue that is highly comparable to normal glandular tissue, and the score 5 will be given to a tumor that is exceedingly undifferentiated.

These 2 scores are added together to get the GS, which is based on a scale from 2 to 10 that indicates how aggressively cancer cells in a sample were observed under a microscope. Tumors in grades 2 through 6 are often slow-growing and have a minimal propensity to spread further, while tumors in grades 7 through 10 are considered to be particularly aggressive.

There are certain drawbacks to Gleason's classification, though. Grades 1 and 2 are extremely uncommon. Hence, the GS has the lowest value of 6, which can be confusing because grade 1 is the lowest grade for other tumors. Additionally, a GS of 7 can be obtained from either a 3 + 4 or obviously a 4 + 3, 2 circumstances with quite different prognoses.^15,16^

Due to these factors, the International Society of Urological Pathology proposed a new classification system in 2016 called GG or Prostate Cancer Grade Group. This system, which derives directly from the GS with the aim of streamlining its reading and improving the prognostic result, has been gradually introduced into clinical practice.

Prostate cancers are categorized using this system into 5 prognostic degree groups. Group 1 comprises all scores below 6, which are actually very uncommon, and Gleason's 6 (3 + 3). While grade 3 indicates prostate tumors with Gleason 4 + 3, grade 2 correlates to Gleason 3 + 4. Groups of grades 4 and 5 correspond to the GS of 8 and Gleason scores of 9 and 10, respectively.^17^

The group with the highest prognostic grade correlates to the lowest grade, and this obviously simplifies prostate cancer classification and staging (Table 2).

### Key Sequences for Prostate Staging

A simple protocol should ideally be used with 3 T equipment to decrease the signal-to-noise ratio, shorten the acquisition time, and keep an acceptable level of spatial resolution. But all of these variables can be updated and optimized by 1.5 T equipment. The endorectal coil is no longer advised due to the patient's discomfort.^14,18,19,20^ In the proposed protocol, a dynamic sequence after intravenous contrast agent (CED) administration and diffusion-weighted imaging (DWI) with *b* values of 0-1000 s/mm^2^ is combined with a high-resolution T2W sequence acquired over 2 planes, including at least one axial (Table 3).^14,19^ The results of a few studies on DWI with an ultra-high *b* value (more than 1000 s/mm^2^) indicated that it might be a promising method for identifying malignant tumors. For example, Bittencourt et al^21^ found that DWI with a value of 1400 s/mm^2^ showed benefits in increasing rates of prostate cancer diagnosis.

To obtain a more appropriate and accurate evaluation of the imaging during synchronous sequence scrolling, it must be emphasized that each sequence must be acquired with precise scanning settings (angle, field of view (FOV), slice thickness, and voxel).^22^ An axial plane T1WI sequence stretching FOV to the aortic bifurcation must be acquired in order to assess nearby lymph nodes and bone as well as its efficacy in detecting postbiopsy prostate hemorrhage (which can easily mimic the low-signal T2 intensity of prostate cancer). The local T-staging of prostate cancer still requires the T2WI pattern.^14^ A fast-spin-echo or turbo-spin-echo to a layer thickness of 3 mm (no-gap) would also be suitable in addition to the aforementioned sequences to assess lymph nodes, vascular-nerve bundles, seminal vesicles, extraprostatic extension, and any bone metastases.^23^ A T2WI 3D acquisition with isotropic voxel and layer width of 1 mm would also be beneficial for a more accurate examination of the aforementioned anatomical structures near to the prostate.^14^

As bone involvement is only detectable during the most advanced stages of the disease with bone scintigraphy (BS), recent studies have indicated that whole-body MRI (WB-MRI) may also have a better diagnostic value than BS.^24^ The PI-RADS guidelines specifically suggest the T1WI, fat-suppressed T2W or short tau inversion recovery, and DWI sequences for the assessment of metastases in WB-MRI.^25,26^

Whole-body-MRI has a diagnostic promise, but there are still some restrictions on how it can be used to stage prostate cancer. The high prices and lengthy scan times are the main causes of these restrictions.

### Magnetic Resonance Imaging Characteristics of Extracapsular Extension

The main objective of local staging of the disease, in accordance with the guidelines of the European Association of Urology (EAU), is to distinguish between a disease that is confined to the gland and a locally advanced one. It is recommended that only patient groups with intermediate or high risk should participate in local illness staging (T2-T3a-T3b). An increased risk of a positive surgical margin (predisposing to recurrence), extracapsular disease dissemination, invasion of the seminal vesicles, lymph node, and distant metastases are all associated with a poorer outcome.^14^ Therefore, it is clear that the decision regarding the type of medication to be given is directly influenced by the information obtained from imaging (radical prostatectomy, radiotherapy, or hormonal deprivation).

Nowadays, there are no specific scores for the locoregional staging of prostate cancer.

A recent study by Mehralivand et al^11^ identified a new score called Extra Prostatic Extension (EPE) GRADE.^11^ Imaging features were implemented in an EPE grading system as follows: grade 0, no suspicion for pathologic EPE; grade 1, either curvilinear contact length or capsular irregularity and bulge; grade 2, both curvilinear contact length and capsular irregularity and bulge; grade 3, frank EPE visible at MRI or invasion of adjacent anatomic structures.

However, these results are being validated. Although the PI-RADS recommendations do not assign a probability of extraprostate extension based on a combination of these results, they do advise that you report these characteristics when analyzing prostate MRI examinations.^11^

**Figure 1.**
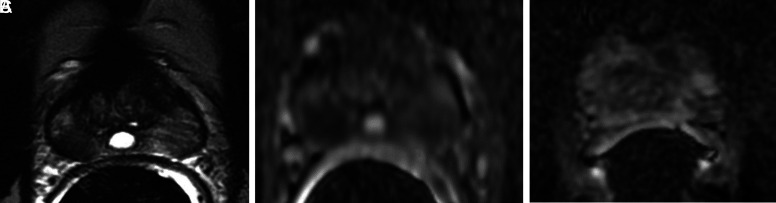
Magnetic resonance images of axial sequences T2WI (A), ADC (B), and DWI (C) of large tumor contact. The prostate gland shows a tumor extension with large contact: in the right peripheral postero-lateral portion of the prostate gland, there is a hypointense lesion in T2WI with wide contact with the capsule without irregularities thereof or without signs of macroscopic extracapsular extension. The lesion is all contained within the capsule and can be classified into a T2 stage. ADC, apparent diffusion coefficient; DWI, diffusion-weighted imaging.

**Figure 2.**
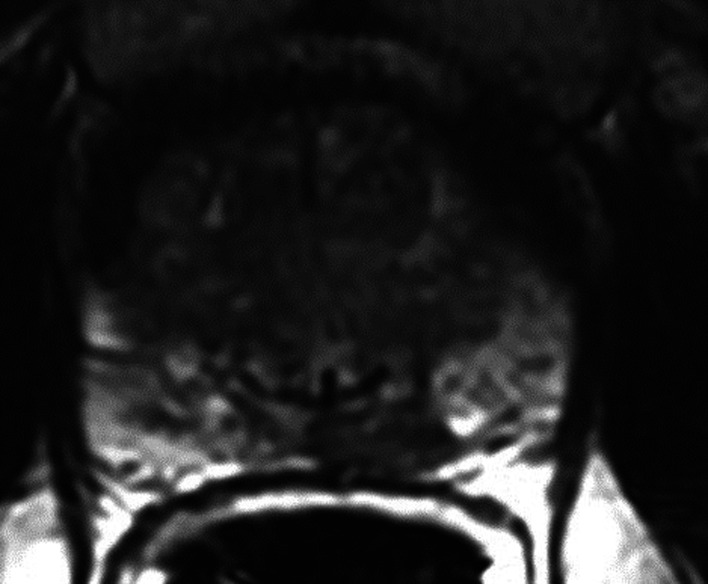
Magnetic resonance image of axial sequences T2WI of the capsular bulging. This alteration is localized to the peripheral region, in the posterior left paramedian seat, with low-signal intensity in T2 and preserved capsular profile, not irregular, but which determines however capsular bulging. Capsular bulging is a lesion that causes a kind of protuberance and begins to exert pressure on the capsule however without infiltrating it from the macroscopic point of view or not trespassing in the extracapsular region.

**Figure 3.**
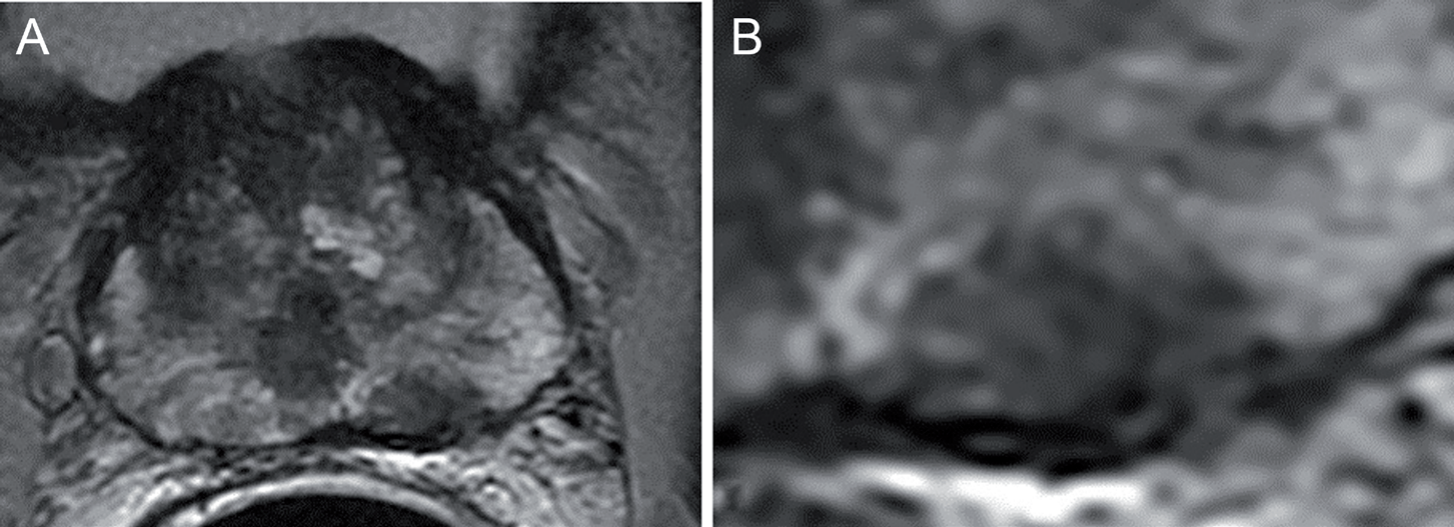
Magnetic resonance images of axial sequences T2WI (A, B) of interrupted capsule. A tumor alteration hypointense exists in the left mid-basal posterior paramedian peripheral zone. In B, we can see better how the capsular profile, represented by a thin hypointense line on the left in the area of contact with the lesion, is interrupted.

**Figure 4.**
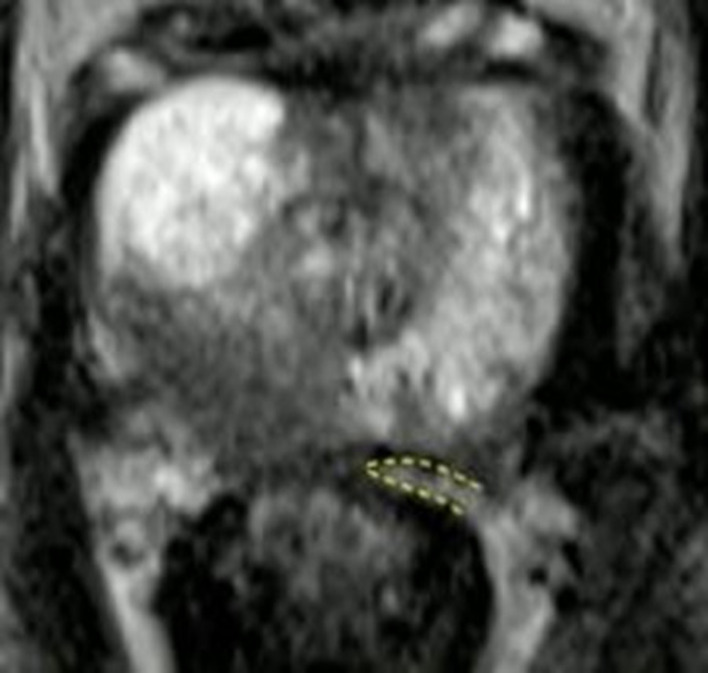
Recto-prostatic angle obliteration is seen in the axial sequences T2WI magnetic resonance imaging. The disappearance of the adipose angle depends on the tumor burden. DWI, diffusion-weighted imaging.

**Figure 5.**
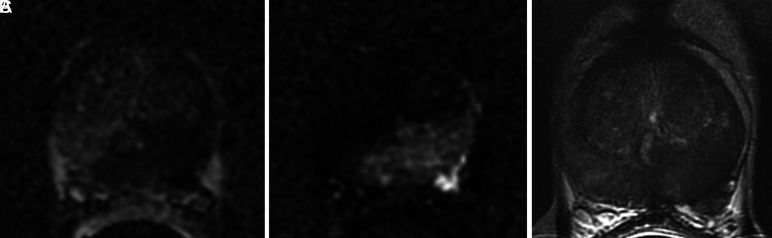
Magnetic resonance images of axial sequences ADC (A), DWI (B), and T2WI (C) of obliteration of the recto-prostatic angle. There is evidence of a large lesion that infiltrates the periprostatic fat in the peripheral area, more evident on the left side with a solid token in the left postero-lateral region. The lesion shows marked hyperintensity in DWI and marked hypointensity in the ADC map. ADC, apparent diffusion coefficient; DWI, diffusion-weighted imaging.

The characteristics of MRI of extraprostatic extension are as follows:

- large tumor contact (Figure 1);
- capsular bulging (Figure 2);
- irregularity/interruption of capsule (Figure 3);
- obliteration of the recto-prostatic angle (Figure 4);
- asymmetry of neurovascular bundles;
- invasion of periprostatic fat (Figure 5); and
- invasion of seminal vesicles.

In clinical practice, it may be useful to consider the results of Baco et al’s study^27^ because there is a close relationship between the contact of prostate cancer with the capsule and the likelihood of extraprostatic disease; in particular, for a contact up to 10 mm, the probability of extraprostatic disease is below 10%, when the contact is 15 mm, then the probability rises around 40%.

If the tumor can be classified as a T3b, it is crucial to consider how the lesion interacts with the seminal vesicles. Since the seminal vesicles and the base of the prostate gland are in close proximity to one another, it is conceivable that the seminal vesicle base will be most affected by penetrated tumors at the base.

**Figure 6.**
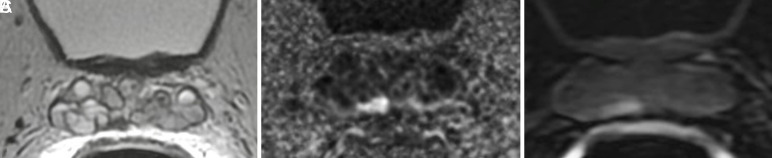
Magnetic resonance images of axial sequences T2WI (A), DWI (B), and Dynamic Contrast Enhanced (DCE) (C) of infiltration of seminal vesicles. Focal parietal thickening of a single seminal vesicle in the right, with low-signal intensity in the T2WI sequences; images of diffusion and perfusion, along with a hyperintensity in DWI and an early enhancement in the T1WI axial picture after contrast, support the result that we have shown in T2. DWI, diffusion-weighted imaging.

**Figure 7.**
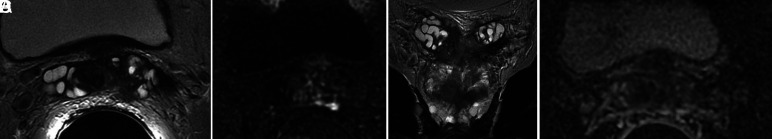
Magnetic resonance images of axial sequences T2WI (A), DWI (B), ADC (C), and coronal sequences T2WI (D) of infiltration of seminal vesicles. Extensive lesion affects the 2 prostate lobes with an intraluminal mass in both seminal vesicles showing a hypointense signal in T2WI. In the coronal image, we see better the obliteration of the vesicular-prostatic angle that represents a specific sign of infiltration of the seminal vesicles. Also, in this case, it is necessary to study not only the T2WI sequences but also DCE and especially the DWI that can help to detect the invasion of seminal vesicles in small infiltrations that present themselves as shaded hypointensity on the T2WI sequences. DWI, diffusion-weighted imaging.

The diagnostic criteria used to establish infiltration of seminal vesicles (Figures 6 and 7) are as follows:

- diffuse wall thickening;
- parietal focal thickening;
- intraluminal mass;
- obliteration of vesicular-prostatic angle;
- structural change;
- restricted diffusion within the lumen of seminal vesicle; and
- postcontrast alteration enhancement.

Another anatomical structure that must be carefully studied is the neurovascular bundle that decorates posterolaterally and bilaterally to the prostate gland. The role of the urologist becomes fundamental, especially in young patients undergoing surgery for prostate adenocarcinoma. Today there are significant improvements in both disease-free survival and quality of life in terms of urinary continence and sexual potency.

**Figure 8.**
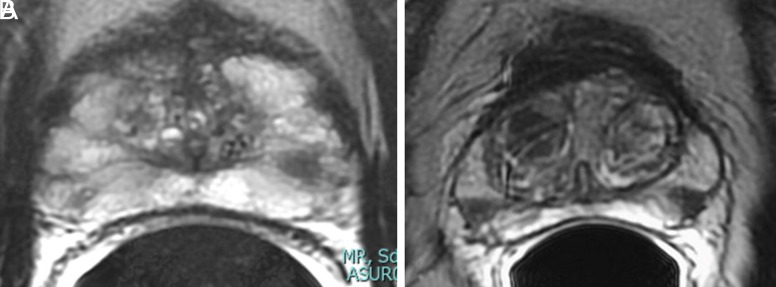
Magnetic resonance images of axial sequences T2WI (A, B) of tumor infiltration of the neurovascular bundle. Nodular lesion far (A) and close (B) to the neurovascular bundle.

“Nerve-sparing” surgery is a surgical technique that aims to remove the prostate by saving 2 bundles of nerves that flow to the sides of the gland and are directed to the penis. These nerves are responsible for erection. The purpose of “Nerve-sparing” surgery is to allow the patient to have negative margins after removal of the tumor and, at the same time, preserve sexual and urinary function while preserving the neurovascular bundle. For these reasons, it is necessary to be as precise as possible in establishing whether or not the tumor lesion infiltrates the neurovascular bundle (Figure 8).^28^

An important problem in the local staging of prostate cancer by MRI was demonstrated by a recent meta-analysis performed on almost 10 thousand patients. This study shows the sensitivity of MRI to extraprostatic disease (T3) is only 57% compared to a specificity of 91%. This is due to the incapacity of MRI to detect microscopic disease.^29^

The MRI of the prostate is also constrained by intrinsic patient features. An organ's signal strength, which affects the quality of the image obtained, is inversely proportional to its distance from the MR system's receiving coil. Since there is an increased distance between the receiver coil and the prostate in individuals with significant obesity, the quality of the study may deteriorate to the point where it is frequently useless for diagnosing the patient's condition. Metal foreign bodies, in particular hip endo-prosthesis (whose prevalence rises with age), are significant factors that impair the effectiveness of the mpMRI test. An accurate evaluation of the mpMRI research may even be impossible due to field distortion brought on by a metal endo-prosthesis.^30^

The guidelines identify prostate cancer as a collector of pathologies with totally different prognoses. The EAU guidelines^31^ and NCCN^32^ agree to identify 3 risk categories (Table 4):

- low;
- intermediate; and
- high.

So you have to consider staging in each risk category because each of them has different clinical priorities. Therefore, in order to give a more reliable answer, it is necessary to know the prevalence of the event. The goal is to minimize overtreatment in patients who are candidates for active surveillance.

In patients with the low clinical risk, it should be remembered that the forewarning of extraprostatic disease is very low (10%-15%).^33^

The EAU guidelines say that it is not necessary to perform a CT for the lymph nodes, and you should not do scintigraphy for the study of bone because the prevalence of pathological lymph nodes is < 10% and so also for bone metastases.^31^

The intermediate risk includes patients with PSA between 10 and 20, a GS of 7, and palpable disease. American guidelines divide intermediate risk into favorable and unfavorable risk. It is a category of patients that is invested with different resources in terms of surgery and radiotherapy, and yet about 30% of these patients will have a recurrence. So, the therapeutic goal is to achieve oncological radicality.

The prevalence of extraprostatic disease in this group of patients rises to 20%-45%.^33^

For this reason, the radiological report must be as precise as possible by marking every area of contact of the tumor with the prostate capsule or bulging to better guide the therapeutic approach.

Patients with adverse intermediate risk need to make a systemic staging, evaluating, with a cross-sectional technical, lymph node by CT and bone metastases by scintigraphy.

Patients who have metastases are included in the high-risk group (PSA > 20 and GS > 7), and the clinical goal is to increase survival.

For men with high-risk prostate cancer who are worried about recurrence, the integration of Positron Emission Tomography (PET) and MRI scanners has opened up new possibilities for multimodal imaging, notably in the diagnosis of nodal and metastatic disease. While ^18^F-choline, ^11^C-choline, ^11^C-acetate, and ^18^F-fluciclovine have all shown promise in prostate cancer imaging, prostate-specific membrane antigen (PSMA) has shown the most promise when combined with MRI. For patients at high risk and those who are worried about a biochemical recurrence, PSMA has shown potential in the diagnosis of both localized and metastatic illness.^34^

Ultra-small superparamagnetic iron oxide-enhanced MRI is one of the new imaging modalities that has demonstrated encouraging results, mostly in the context of clinical research. To evaluate the clinical value of such agents, more research will be needed.^35^

Many studies show how the next-generation imaging techniques that combine the morphological data with the functional like the WB-MRI or the PET CT are excellent in the staging of patients at high risk and allow a better layering of the patient and therefore the choice of the best therapy. The phases of prostate cancer progression may need to be redefined if previously undetectable metastases are found using next-generation imaging techniques. This means that early or locally targeted systemic treatment can improve the patient's outcomes.

## Conclusion

Magnetic resonance imaging has a very important role in promoting precision oncology because it can respond to the needs of individual patients. The needs of patients with prostate cancer are very different. The low-risk patient must preserve the function and therefore sexual power and continence. Those with intermediate risk have the need to eradicate the disease and avoid recurrence. Finally, the patient with high risk has as its objective the increase of survival and therefore it is necessary to search for small metastases in order to avoid useless treatments.

The use of mpMRI, integrated with patient clinical data and next-generation imaging techniques, allows you to benefit from the latest technological advances, such as robotic surgery or stereotactic radiotherapy, with the aim of obtaining a personalized medicine, integrating clinical data with imaging for a successful therapeutic strategy.

Therefore, MRI is definitely a diagnostic tool in exclusively radiological hands that tends to play an increasingly important role in the diagnostic algorithms of prostate cancer.

## Figures and Tables

**Table 1.** Tumor, Node, Metastasis Staging System

T—Primary tumor	TX Primary tumor cannot be assessed	T0 No evidence of primary tumor	T1 Clinically inapparent tumor that is not palpable T1a: Tumor incidental histological finding in 5% or less of soft tissue resected T1b: Tumor incidental histological finding in more than 5% of soft tissue resected T1c: Tumor identified by needle biopsy	T2 Tumor that is palpable and confined within the prostate T2a: Tumor involves one-half of one lobe or less T2b: Tumor involves more than half of one lobe, but not both lobes T2c: Tumor involves both lobes	T3 Tumor extends through the prostatic capsule^a^ T3a: Extracapsular extension (unilateral or bilateral), including microscopic bladder neck involvement T3b: Tumor invades seminal vesicle(s)	T4 Tumor is fixed or invades adjacent structures other than seminal vesicles: external sphincters, rectum, levator muscles, and/or pelvic wall	N—regional lymph nodes^b^	NX regional lymph nodes cannot be assessed	N1 no regional lymph node metastasis	N1 regional lymph node metastasis	M—distant metastasis^c^	M0 no distant metastasis	M1 distant metastasis M1a: nonregional lymph node(s) M1b: bone(s) M1c: other site(s)

^a^Invasion into the prostatic apex or into (but not beyond) the prostatic capsule is not classified as T3 but as T2.

^b^Metastasis no larger than 0.2 cm can be designed pNmi.

^c^When more than one site of metastasis is present, and the most advanced category is used. (p)M1c is the most advanced category.

**Table 2.** Comparison Between Gleason Score (GS) and the New ISUP Grade Group (GG)

Gleason Score	ISUP Grade Group
2–6	1
7 (3 + 4)	2
7 (4 + 3)	3
8 (4 + 4 or 3 + 5 or 5 + 3)	4
9–10	5

ISUP, International Society of Urological Pathology.

**Table 3.** Prostate Imaging Reporting and Data System v2.1 Recommended MR Imaging Protocol

Imaging Sequence	Technical Parameters
T2WI	Axial plane and a minimum of one additional orthogonal plane (either sagittal or coronal)
	Straight axial plane to the patient or to the long axis of the prostate	In-plane resolution: ≤ 0.7 mm (phase) × ≤ 0.4 mm (frequency)	Slice thickness/gap: 3 mm/0 mm	FOV: 12–20 cm to image the entire prostate gland and seminal vesicles
	DWI	Axial plane (same locations as for T2WI)
		In-plane dimension: ≤ 2.5 mm phase and frequency	FOV: 16–22 cm	TE: ≤ 90 ms; TR: > 3000 ms	Section thickness/gap: 3 mm/0 mm	Free-breathing spin echo EPI sequence combined with spectral fat saturation is recommended	ADC map calculation: low b value should be set at 0–100 s/mm^2^, high *b* value should be < 1000 s/mm^2^	“High *b* value”: *b* value of ≥ 1400 s/mm^2^; it can be acquired by scanning or calculated
		DCE	Axial plane (same locations as for T2WI)
Fat suppression and/or subtraction is recommended			2D or 3D T1 GRE sequence (preferred)	Section thickness/gap: 3 mm/0 mm	Injection rate: 2–3 mL/s	TR/TE: <100 ms/< 5 ms	In-plane dimension: ≤ 2 mm × ≤ 2 mm	Temporal resolution: B 15 s	Total observation: > 2 min

2D, two-dimensional; 3D, three-dimensional; ADC, apparent diffusion coefficient; DW, diffusion weighted; EPI, echo planar imaging; FOV, field of view; GRE, gradient echo; MR, magnetic resonance; T2W, T2 weighted; TE, echo time; TR, repetition time.

**Table 4.** European Association of Urology Risk Groups for Biochemical Recurrence of Localized and Locally Advanced Prostate Cancer

Definition			
Low Risk	Intermediate Risk	High Risk	
PSA < 10 ng/mL	PSA 10-20 ng/mL	PSA > 20 ng/mL	Any PSA
GS < 7 (ISUP grade 1) and cT1-2a	GS 7 (ISUP grade 2/3) or cT2b	GS > 7 (ISUP grade 4/5) or cT2c	Any GS (any ISUP grade) cT3-4 or cN+
**Localized**			**Locally advanced**

GS, Gleason score; ISUP, International Society for Urological Pathology; PSA, prostate-specific antigen.
